# A Prospective Study of Eplerenone in the Treatment of Patients with Glomerulonephritis

**DOI:** 10.3390/biomedicines11123340

**Published:** 2023-12-18

**Authors:** Marios Papasotiriou, Georgia Andrianna Georgopoulou, Adamantia Mpratsiakou, Theodoros Ntrinias, Georgios Lyras, Dimitrios S. Goumenos, Evangelos Papachristou

**Affiliations:** Department of Nephrology and Kidney Transplantation, University Hospital of Patras, 26504 Patras, Greece; up1081257@upatras.gr (G.A.G.); med5319@ac.upatras.gr (A.M.); th.ntrinias@gmail.com (T.N.); glyras1506@gmail.com (G.L.); dgoumenos@upatras.gr (D.S.G.); epapachr@upatras.gr (E.P.)

**Keywords:** eplerenone, glomerulonephritis, proteinuria, blood pressure

## Abstract

Background: High aldosterone levels contribute to kidney disease progression, while spironolactone in combination with ACEi or ARBs can potentially reduce proteinuria and ameliorate kidney function deterioration. However, evidence on the impact of eplerenone in patients with glomerulonephritis is scarce. Methods: In this prospective observational study, we assessed the effects of eplerenone in patients with biopsy-proven glomerulonephritis who were already treated with ACEi or ARBs. Patients received either eplerenone (25 mg daily) on top of ACEi or ARBs or standard treatment alone. Proteinuria (24 h total protein excretion), kidney function, blood pressure and serum K^+^ levels were assessed at 3, 6 and 12 months after the initiation of treatment. Results: Sixty-six patients were included in the study. Eplerenone was administered in 30 patients, while 36 received only ACEi or ARB. Proteinuria decreased from 1768 to 1152 mg/24 h after 1 year of eplerenone treatment, while it remained stable in controls. Eplerenone showed significant impact on proteinuria in those with baseline proteinuria of >1000 mg/24 h. Patients who received eplerenone showed a reduction in systolic blood pressure, while eGFR and serum K^+^ levels remained stable. Conclusions: Addition of eplerenone has a beneficial effect on proteinuria in patients with glomerulonephritis and significant baseline proteinuria.

## 1. Introduction

Aldosterone is a mineralocorticoid hormone that is up-regulated by angiotensin II, high serum potassium (K^+^) levels and adrenocorticotropic hormone [1,2]. Aldosterone regulates extracellular fluid volume and K^+^ metabolism by increasing sodium reabsorption in the principal cells of the distal nephron. Sodium reabsorption is mediated via activation of the apical epithelial sodium channel (ENaC) and the basolateral Na^+^- K^+^ ATPase, and aldosterone promotes the expression of the gene that encodes the a-subunit of ENaC [2]. Apart from these classical effects, aldosterone has a deleterious impact on the heart, the vasculature and the kidney by promoting vascular remodeling, collagen formation, endothelial dysfunction and organ fibrosis overall [3,4,5].

Primary aldosteronism has been acknowledged as a highly prevalent but under-recognized entity in hypertensive as well as normotensive individuals [6], and excess aldosterone production conveys a higher risk of damage in target organs, such as the cardiovascular system and the kidneys [7,8]. Moreover, experimental and clinical evidence suggest that aldosterone contributes to progressive kidney disease [8,9,10]. Numerous studies have demonstrated that activation of the renin–angiotensin–aldosterone system (RAAS) has a detrimental effect on the progression of CKD and inhibition of the RAAS with angiotensin-converting enzyme inhibitors (ACEIs) or angiotensin type 1 receptor blockers (ARBs) can delay disease progression [11,12]. Interestingly, plasma aldosterone levels may rebound and increase to a significant extent following an initial decline after administration of ACEi or ARBs [13]. This phenomenon, known as ‘aldosterone breakthrough’, could have important clinical consequences given aldosterone’s pro-fibrotic actions on the kidneys, especially in patients with established CKD. Depending on the definition used, the incidence of aldosterone breakthrough extends over a wide range from 10% after 6 months to 53% after 1 year of ACEi or ARB treatment. Nevertheless, breakthrough definition is somehow problematic, as in some studies, it is defined as any increase from an individual’s baseline serum aldosterone level, while others set an absolute cutoff value of serum aldosterone. The latter studies have detected lower risks of breakthrough [13]. In any case, a large proportion of patients with CKD already on ACEi or ARB treatment could potentially benefit from mineralocorticoid receptor (MR) antagonist add-on therapy. Indeed, treatment with MR antagonists, especially spironolactone, has been shown to have favorable effects in patients with CKD of various etiologies by reducing albuminuria and the degree of estimated glomerular filtration rate (eGFR) decrease [14]. Even treatment with the non-steroidal MR antagonist finerenone decreased the risk of CKD progression in patients with CKD and diabetes in the recent FIDELIO-DKD and FIGARO-DKD trials [15,16]. Nevertheless, data on safety and the efficacy of eplerenone on CKD progression and proteinuria reduction, especially in patients with glomerulonephritis, are scanty.

In this prospective observational study, we evaluated the effects of the MR antagonist eplerenone on proteinuria and kidney function (expressed as eGFR) in a cohort of patients with biopsy-proven GN who were treated with eplerenone (25 mg/d) for 1 year.

## 2. Materials and Methods

In this prospective observational cohort study, we evaluated the effects of eplerenone on proteinuria and eGFR as well as its safety in patients with biopsy-proven glomerulonephritis. All patients with biopsy-proven glomerulonephritis treated and actively monitored in our department’s outpatient clinic were screened during a 3-month period. All patients were offered treatment with eplerenone for 1 year on top of their standard treatment, and those who agreed were prescribed 25 mg of eplerenone once daily. Those who did not agree to receive eplerenone on top of their standard treatment were considered as controls.

Inclusion criteria comprised the following: age > 18 years, previous treatment with ACEi or ARB at maximum tolerated dose for at least 6 months prior to screening, proteinuria greater than 150 mg in a 24 h urine collection, serum potassium less than 5 mg/dL at screening and no evidence of active cancer. We excluded patients with hepatic dysfunction, history of allergy or non-tolerability to ACEi or ARBs, acute kidney injury in the past three months and those on active immunosuppressive treatment with corticosteroids alone or in combination with calcineurin inhibitors, anti-metabolites or monoclonal antibodies. Antihypertensive drugs apart from ACEi or ARBs were prescribed as needed to achieve a target systolic blood pressure (SBP) of less than 120 mmHg. Dosage of ACEIs and ARBs remained stable after inclusion in the study. No patient received treatment with an SGLT-2 inhibitor prior to or during the study period.

All patients were advised to follow a low-protein (approximately 0.8 g/kg/day) and low-salt (4–5 g NaCl/day) diet. Moreover, patients who received eplerenone received comprehensive dietary counseling on a low-K^+^ diet and avoidance of high K^+^ content fruits and vegetables. We did not measure urinary Na^+^ and K^+^ excretion to assess dietary compliance. If serum K^+^ levels increased above the threshold of 5.5 meq/L, eplerenone was discontinued.

Baseline evaluation included standard office BP measurements as suggested by the European Society of Hypertension [17], eGFR calculation using the CKD-EPI equation and measurement of 24 h urine protein and plasma aldosterone levels. Thereafter, patients were followed in the outpatient clinic after 3, 6 and 12 months. Blood pressure and heart rate were measured three times at each visit, and a blood sample was drawn after the patient had remained seated for 30 min. At each clinic visit, including the baseline evaluation, each subject was asked to bring a 24 h urine collection for urine protein measurement.

The Human Research Committee of the University Hospital of Patras approved the study protocol (number of approval: 7251/14.12.20), and all subjects provided their written informed consent prior to inclusion in the study. The study protocol is in accordance with the Helsinki Declaration as revised in 2013.

## 3. Aldosterone Breakthrough

All patients included were screened for the presence of the aldosterone breakthrough phenomenon by measuring plasma aldosterone levels at enrollment, which was preceded by at least 6 consecutive months of ACEi or ARBs treatment. Blood was collected between 8:30 and 10:00 a.m., after the patient had remained seated for 30 min. After collection, specimens were centrifuged, and plasma was stored at −80 °C. Measurements were performed using a commercially available radioimmunoassay kit according to manufacturer instructions, and normal values of aldosterone ranged between 50 and 175 pg/mL. Aldosterone breakthrough was defined as aldosterone levels greater than 175 pg/mL.

## 4. Statistical Analysis

Continuous variables are presented as means with standard deviations (SD) and range of values as appropriate, while qualitative variables are presented with frequencies and percentages. The Kolmogorov–Smirnov test was used for normality analysis of the continuous variables. Normally distributed and skewed data were compared with the simple *t*-test and the Mann–Whitney test, respectively, for investigating the differences of means between baseline or final continuous data of patients who received eplerenone and standard treatment alone (control group) or to investigate the differences of means at each time point between the two groups. In order to investigate the differences between the means at multiple time points in separate groups, we used repeated measures ANOVA with Bonferroni post-hoc tests for normally distributed data or Friedman with Dunn’s post-hoc tests for skewed data. A mixed effects model repeated measures analysis was used in cases of missing values at specific time points.

For statistical analyses, we used SPSS for Windows (version 16.0 SPSS Inc., Chicago II, USA) or GraphPad Prism (version 8.0.2 for Windows, GraphPad Software, San Diego, CA, USA).

## 5. Results

Overall, 87 patients with biopsy-proven glomerulonephritis were screened, and 66 were included in the final analysis. Sixteen patients were excluded due to concurrent immunosuppressive treatment, and other exclusions included two due to high serum potassium levels, one due to recent acute kidney injury, one due to nephrotic syndrome relapse just prior to eplerenone assignment (imminent commencement of immunosuppressive treatment) and one due to absence of prior treatment with ACEi/ARB. The distribution of patients among the various types of glomerular disease was as follows: 25 had membranous nephropathy, 19 had IgA nephropathy, 12 had focal and segmental glomerulosclerosis, four had minimal change disease, four had ANCA-associated vasculitis, one had lupus nephritis class V and one had IgM nephritis. At the time of inclusion, all patients had been followed in the outpatient clinic for at least 1 year, and none had received a combination of ACEi plus ARBs. Thirty patients agreed to receive eplerenone and were assigned to the intervention arm of the study, while 36 patients continued their standard treatment with either an ACEi or an ARB. Twenty patients in the eplerenone group were treated with an ARB, and 10 were treated with an ACEi, while in the standard treatment group, 27 subjects were treated with an ARB, and nine were treated with an ACEi. Finally, six patients in the eplerenone group and eight in the control group received hydrochlorothiazide as an anti-hypertensive treatment, while no patient received furosemide. The baseline characteristics of patients treated with eplerenone and those treated with standard therapy are presented in Table 1.

Aldosterone breakthrough phenomenon was present in 18 patients (27.3%) overall. Ten of them belonged to the intervention arm, and the remaining eight belonged to the standard therapy arm.

## 6. Proteinuria

**Figure 1:**
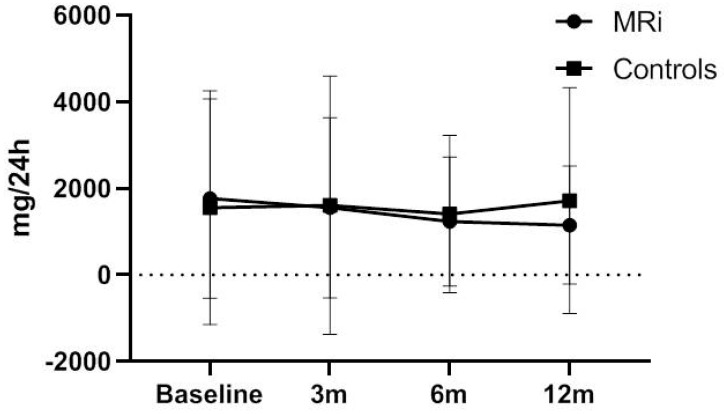
Proteinuria progression in patients treated with eplerenone and controls. m: months from the initiation of treatment, MRi: mineralocorticoid receptors inhibitors.

Baseline proteinuria was 1768 ± 2303 mg/24 h in patients treated with eplerenone and 1557 ± 2702 mg/24 h in patients treated with conventional therapy. After 1 year of follow-up, proteinuria decreased to 1152 ± 1363 mg/24 h among patients treated with eplerenone, but it did not change among patients treated with conventional therapy (Table 2). Reduction of proteinuria was already detectable at 3 months after initiation of treatment in patients who received eplerenone and followed a constant decrease slope throughout follow-up (Figure 1). However, there were no significant differences in proteinuria between the two groups of patients at all time points of follow-up, as presented in Supplemental Table S1.

Subdivision of the active treatment arm for patients with proteinuria greater or lesser than 1000 mg/24 h showed that only the former cohort benefited from eplerenone, while patients with lesser proteinuria had no significant change (Table 3). Nevertheless, four patients who received eplerenone showed nephrotic syndrome relapse during follow-up.

For patients who were diagnosed with aldosterone breakthrough, subgroup analysis revealed that proteinuria decreased from 822.8 ± 910.6 mg/24 h to 321.5 ± 360.4 mg/24 h (*p* = 0.05) at 12 months after initiation of eplerenone. In contrast, patients who received ACEi or ARBs alone showed unchanged proteinuria levels at the end of follow-up (from 912 ± 611.5 to 951 ± 787.7 mg/24 h).

## 7. Kidney Function

**Figure 2:**
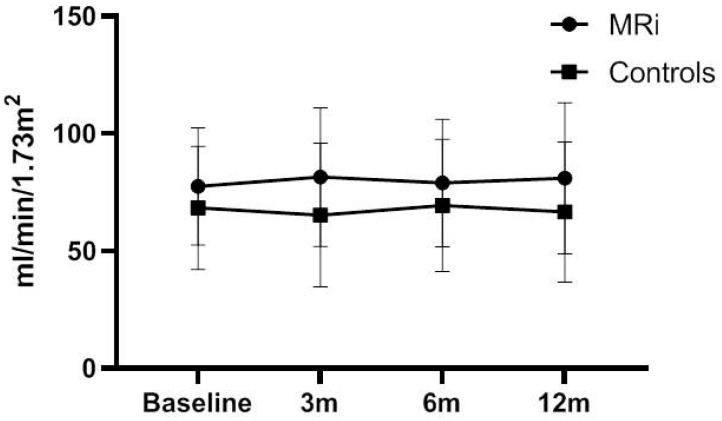
Estimated GFR progression in patients treated with eplerenone and controls. m: months from the initiation of treatment, MRi: mineralocorticoid receptors inhibitors.

After 3 months of treatment with eplerenone, we observed no significant change in serum creatinine and eGFR. This trend of serum creatinine and eGFR preservation was also documented at both 6 and 12 months after initiation of treatment. Data concerning serum creatinine and eGFR in both groups of patients are presented in Table 4 and Figure 2. Differences of kidney function parameters between groups at different time points are presented in Supplemental Table S1.

Patients in the eplerenone arm, either with baseline eGFR < 60 or eGFR ≥ 60 mL/min/1.73 m^2^, experienced a non-significant change in eGFR after initiation of treatment (from 49.6 ± 5.7 to 52.5 ± 8.7 mL/min/1.73 m^2^ and from 91.2 ± 18.9 to 92 ± 22.8 mL/min/1.73 m^2^, respectively).

Baseline kidney function did not differ significantly among patients with aldosterone breakthrough in the active treatment arm and the control group. However, kidney function remained stable at 12 months after initiation of treatment (serum creatinine from 1.06 ± 0.38 to 1.03 ± 0.42 mg/dL, *p* = 0.43) in those who received eplerenone, whereas patients who received standard treatment showed a non-significant kidney function deterioration with an increase in serum creatinine levels from 1.44 ± 0.71 to 1.73 ± 1.17 mg/dL (*p* = 0.3).

## 8. Blood Pressure

**Figure 3:**
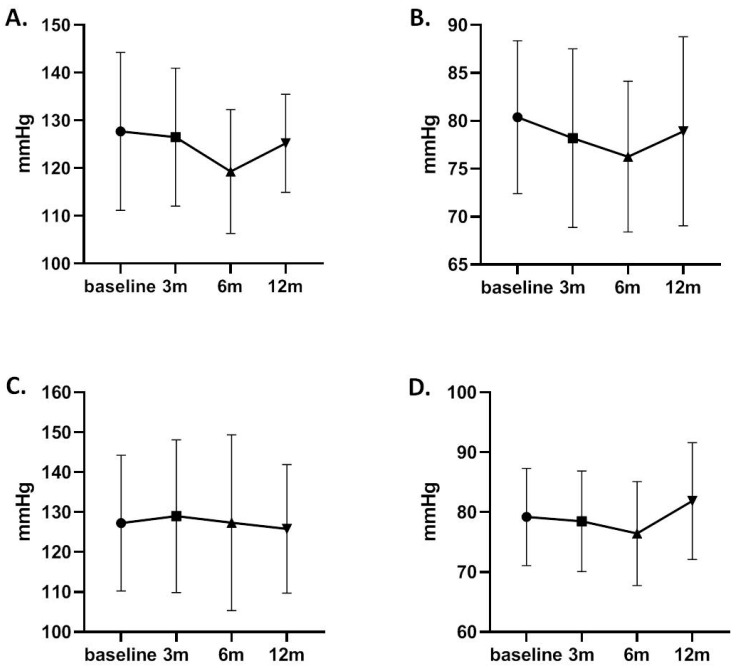
Systolic (**A**) and diastolic (**B**) blood pressures in patients treated with eplerenone and systolic (**C**) and diastolic (**D**) blood pressures in controls. m: months from the initiation of treatment.

Baseline systolic blood pressure (SBP) and diastolic blood pressure (DBP) in the eplerenone arm were 127.7 ± 16.6 mmHg and 80.4 ± 8 mmHg, respectively, while in patients who received standard treatment, SBP and DBP were 127.3 ± 17 and 79.2 ± 8.1 mmHg, respectively. After 6 and 12 months of treatment with eplerenone, SBP decreased significantly (*p* = 0.037) to 119.3 ± 13 and 125.2 ± 10.3 mmHg, respectively, while a minor non-significant decrease was observed in patients who received conventional treatment. Diastolic BP at 12 months of treatment showed a non-significant decrease in the intervention arm, but it was significantly increased in the control group (Table 5 and Figure 3). Overall, BP did not show significant differences between the two groups of patients at the end of follow-up as well as at all other time points, as presented in Supplemental Table S1.

Baseline aldosterone levels did not seem to affect BP, as those with aldosterone breakthrough showed no significant differences in SBP and DBP in comparison to patients with lower plasma aldosterone levels (SBP: 128.7 ± 19.2 vs. 127.1 ± 16.6, *p* = 0.7 and for DBP: 79 ± 6.8 vs. 80 ± 8.9, *p* = 0.6). Overall, in this subgroup of patients, SBP and DBP did not exhibit significant alteration during the follow-up period of 12 months whether on eplerenone or on standard treatment.

## 9. Serum Potassium

Serum K^+^ increased significantly 3 months after initiation of treatment (*p* = 0.04) but remained stable and overall unchanged during the rest of follow-up in the eplerenone arm. On the contrary, serum K^+^ did not show any significant change in patients who received standard treatment (Table 6). Differences of serum K^+^ levels between groups at different time points are presented in Supplemental Table S1. None of the patients in the eplerenone group, irrespective of kidney function, increased their serum K^+^ levels over 5.5 meq/L, and thus this was not a reason for eplerenone discontinuation.

Baseline serum K^+^ did not seem to be affected by aldosterone overproduction, as patients with aldosterone breakthrough and those with lower plasma aldosterone levels had similar K^+^ levels (4.45 ± 0.37 vs. 4.46 ± 0.41, *p* = 0.9). Among patients with aldosterone breakthrough, serum K^+^ was similar among those treated with eplerenone and those on standard treatment. In this group of patients, serum K^+^ increased non-significantly (from 4.3 ± 0.3 to 4.7 ± 0.4 meq/L, *p* = 0.18) after 3 months of eplerenone treatment and remained stable thereafter. Patients with aldosterone breakthrough in the control group had stable serum K^+^ throughout follow-up.

## 10. Adverse Events and Dropouts

During follow-up, five patients presented with relapse of nephrotic syndrome, four of whom were on treatment with eplerenone. Moreover, two patients discontinued treatment after 6 months, the first due to reported hypotension and the second due to upper limb numbness. No cases of gynecomastia were noted in the male participants, and there were no incidents of hyperkalemia overall. Four patients were lost to follow-up (one in the eplerenone treatment arm).

## 11. Discussion

In this prospective study of eplerenone treatment on top of standard treatment with ACEi or ARBs in patients with glomerular disease, we have shown that eplerenone is effective in reducing proteinuria in patients with a higher degree of baseline proteinuria. Moreover, patients who received eplerenone maintained stable kidney function after 12 months, while those who received conservative treatment alone showed a trend of decline in eGFR.

Effectively reducing proteinuria remains the main target of therapy in patients with primary glomerulonephritis, as proteinuria almost invariably correlates with progression to end-stage kidney disease [18]. ACEi or ARB administration remains the cornerstone of antiproteinuric treatment, while sodium glucose transporter 2 inhibitors have recently emerged as an important add-on option [19,20]. Other options include MRAs, either in the form of first-generation spironolactone or the second generation, and, with less adverse effects, eplerenone. Concerning spironolactone, there is evidence that when administered in combination with ACEi or ARBs, it reduces proteinuria more than standard treatment with ACEi or ARBs, even at a dose of 25 mg/day [14]. This feature is also prominent in patients with diabetic kidney disease and albuminuria [21]. Eplerenone has exhibited an antiproteinuric effect in experimental nephrotic syndrome [22], and additionally, it reduces albuminuria effectively in non-diabetic CKD patients after 8 weeks of administration at a dose of 25–50 mg once daily as an add-on treatment to ACEi or ARBs [23]. Higher doses of eplerenone up to 100 mg/day have also shown a significant effect on albuminuria, up to almost 50%, in patients with diabetic nephropathy [24]. Treatment for longer periods, up to 1 year, has also shown benefit in albuminuria in patients with hypertension and CKD [25]. Nevertheless, addition of spironolactone to ACEi did not further reduce proteinuria in one study that included 11 patients with membranous nephropathy and nephrotic syndrome [26]. The beneficial anti-albuminuric effect of eplerenone in the aforementioned studies involved patients in the spectrum of modest albuminuria up to 599 mg/g (urine albumin/creatinine ratio) [25] to higher values of over 1 gr/24 h [23]. In our study, though, significant reduction of proteinuria after eplerenone treatment was seen mainly in patients with baseline proteinuria greater than 1000 mg/24 h, while patients with proteinuria of lesser degree showed a non-significant reduction.

Long-term preservation of kidney function is the ultimate treatment goal for patients with CKD. Previous studies have demonstrated that addition of spironolactone to treatment with ACEi or ARBs initially lowered the eGFR [27] but stabilized kidney function in the long run [14]. eGFR reduction directly after initiation of spironolactone has also been demonstrated in cohorts of patients with type 1 or 2 diabetes and albuminuria [28,29]. Concerning eGFR after eplerenone administration, the same short-term pattern is observed in diabetic as well as non-diabetic patients with CKD and albuminuria. Nevertheless, the early decrease in eGFR is less than 30% from baseline values, and treatment cessation is rarely necessary [23,24]. In our cohort, those treated with eplerenone showed an insignificant increase in serum creatinine 3 months after initiation of treatment, but overall, kidney function was stable during the 12 months of follow-up. On the contrary, patients who received conservative treatment showed a more pronounced but still not statistically significant decline in kidney function at the end of follow-up. Finally, overall kidney function between the two groups at the end of follow-up did not show significant difference.

Addition of spironolactone to ACEi or ARBs has been shown to reduce BP effectively in patients with diabetic as well as non-diabetic CKD and albuminuria [14,29]. Likewise, initiation of eplerenone on top of standard treatment has a significant lowering effect on SBP in patients with diabetic nephropathy or non-diabetic CKD [23,30]. However, all of the aforementioned studies included patients with a target SBP of less than 130 or 125 mmHg, while in our study, the target SBP was 120 mmHg, indicating intensified anti-hypertensive treatment. Concerning DBP, eplerenone appears to be effective in patients with CKD and proteinuria, although data are more inconsistent in patients with diabetic nephropathy and show either an important lowering or neutral effect [23,24,30]. In this study, patients treated with eplerenone showed a significant overall reduction of SBP, which was more apparent 6 months after initiation of treatment. This reduction could have at least a partial positive impact on the proteinuria reduction that was shown in these patients. Diastolic BP, however, was not significantly altered. Interestingly, patients who received only standard treatment also showed a non-significant reduction in SBP, and thus the SBP values between the two groups of patients at the end of follow-up were not significantly different.

High aldosterone levels are independently associated with kidney function deterioration in patients with CKD [9], and treatment with MRAs attenuates the progression of kidney disease [1]. Furthermore, patients with higher aldosterone levels exhibit a greater degree of proteinuria and lower serum potassium [9,14]. These features are also found in patients with aldosterone breakthrough [9,14]. Although the incidence of aldosterone breakthrough can reach 50% of patients after 1 year of treatment with ACEi or ARBs, it was diagnosed in only 29% of patients in our cohort. More importantly, those with aldosterone breakthrough who received eplerenone showed reduction of proteinuria and better maintained kidney function in comparison to those on standard therapy. Nevertheless, this favorable outcome was not accompanied by significant BP reduction in this subgroup of patients.

Adverse effects of treatment with MRAs in patients with CKD include, but are not limited to, hyperkalemia and gynecomastia. While both of these features are prominent in patients treated with spironolactone [14], data on eplerenone prove that this agent is better tolerated with minimal or no treatment withdrawals due to hyperkalemia [23,25]. In our cohort, we detected no cases of significant hyperkalemia, and no patient withdrew from eplerenone treatment due to high K^+^ serum levels. This could be attributed to the comprehensive dietary counseling provided to patients, which ensured a low-K^+^ diet and avoidance of certain vegetables and fruits. Moreover, none of our male patients developed gynecomastia or other pronounced female characteristics, which offers a substantial advantage over traditional treatment with spironolactone. Overall, eplerenone was well tolerated, and only two patients discontinued treatment due to reported low BP and upper limb numbness.

Limitations of our study include its single-center design and its relatively low number of patients who followed active treatment with eplerenone. Moreover, the lack of a placebo-controlled arm may have affected the final results. Furthermore, the significant reduction in SBP in the active treatment arm could have an impact on proteinuria reduction. Nevertheless, this could not be further assessed. Finally, a longer follow-up period could potentially demonstrate the beneficial effect of eplerenone on kidney function or a sustained effect on proteinuria regression more clearly. Prospective randomized placebo-controlled trials with more patients with glomerulonephritis and longer duration of intervention are needed to address this issue.

In conclusion, in this prospective study, we have shown that administration of eplerenone as an add-on treatment to ACEi or ARBs in patients with chronic glomerulonephritis can be beneficial in proteinuria reduction in those with baseline values of more than 1000 mg/24 h with a favorable safety profile.

## Figures and Tables

**Table 1:** Baseline clinical and biochemical characteristics of patients treated with eplerenone and controls.

	Patients Treated with Eplerenone	Controls	*p*
Sex (males/females)	20/10	18/18	0.22
Age (years)	52.8	56.4	0.3
Serum creatinine (mg/dL)	1.08	1.18	0.3
eGFR (mL/min/1.73 m^2^)	77.6	68.4	0.16
Proteinuria (mg/24 h)	1768	1557	0.74
Serum K^+^ (mmol/L)	4.45	4.47	0.89
Systolic blood pressure (mmHg)	127.7	127.3	0.9
Diastolic blood pressure (mmHg)	80.4	79.2	0.59
Histological Diagnosis *N* (%)			
Membranous nephropathy	12 (46.2)	14 (53.8)	-
IgA Nephropathy	9 (47.4)	10 (52.6)	-
Focal Segmental Glomerulosclerosis	6 (50)	6 (50)	-
ANCA vasculitis	0	4 (100)	-
Minimal change disease	2 (50)	2 (50)	-
IgM nephropathy	1 (100)	0	-
Comorbidities (Yes/Νo)			
Diabetes mellitus	1/29	7/29	0.06
Coronary heart disease	4/26	3/33	0.69
Heart failure due to low left ventricular ejection fraction (<45%)	1/29	0/36	0.46
Anti-hypertensive drugs (n ± SD)	1.37 ± 0.62	1.6 ± 0.73	0.15

**Table 2:** Proteinuria progression in patients treated with eplerenone and controls.

	Baseline Proteinuria (mg/24 h)	Proteinuria 3 Months	Proteinuria 6 Months	Proteinuria 12 Months	*p*
Patients treated with eplerenone	1768 ± 2303	1555 ± 2083	1237 ± 1491	1152 ± 1363	0.06
Controls	1557 ± 2702	1613 ± 2982	1411 ± 1819	1718 ± 2611	0.46

**Table 3:** Proteinuria progression according to baseline levels (<1000 vs. ≥1000 mg/24 h) in patients treated with eplerenone and controls.

Patients Treated with Eplerenone	Baseline Proteinuria (mg/24 h)	Proteinuria 3 Months	Proteinuria 6 Months	Proteinuria 12 Months	*p*
Baseline Uprot < 1000 mg/24 h	425.2 ± 270.6	462.9 ± 415.8	378.9 ± 311.8	356.1 ± 300.6	0.418
Baseline Uprot ≥ 1000 mg/24 h	3630.8 ± 2955.2	3082.5 ± 2765.4	1888.2 ± 1823.3	1483.3 ± 1212.1	<0.001
Controls					
Baseline Uprot < 1000 mg/24 h	437.6 ± 326	470.3 ± 427	571.9 ± 562	1097.3 ± 1876	0.709
Baseline Uprot ≥ 1000 mg/24 h	4072 ± 4509	4233.4 ± 4881	2970.8 ± 2525	3357.3 ± 3929	0.795

**Table 4:** Kidney function progression (as expressed with serum creatinine and eGFR) in patients treated with eplerenone and controls.

	Baseline Serum Creatinine (mg/dL)	Serum Creatinine 3 Months	Serum Creatinine 6 Months	Serum Creatinine 12 Months	*p*
Patients treated with eplerenone	1.076 ± 0.31	1.077 ± 0.29	1.092 ± 0.28	1.091 ± 0.3	0.24
Controls	1.179 ± 0.472	1.296 ± 0.557	1.197 ± 0.54	1.292 ± 0.664	0.09
	Baseline eGFR (mL/min/1.73 m^2^)	eGFR 3 months	eGFR 6 months	eGFR 12 months	
Patients treated with eplerenone	77.55 ± 24.9	81.5 ± 29.5	79 ± 27.2	81 ± 32.1	0.8
Controls	68.39 ± 26.2	65.37 ± 30.5	69.46 ± 28.1	66.63 ± 29.8	0.08

**Table 5:** Systolic (SBP) and diastolic (DBP) pressure progression in patients treated with eplerenone and controls.

	Baseline SBP (mmHg)	SBP 3 Months	SBP 6 Months	SBP 12 Months	*p*
Patients treated with eplerenone	127.7 ± 16.6	126.5 ± 14.5	119.3 ± 13	125.2 ± 10.3	0.016
Controls	127.3 ± 17	129.0 ± 19.11	127.4 ± 22	125.8 ± 16.1	0.95
	Baseline DBP	DBP 3 months	DBP 6 months	DBP 12 months	
Patients treated with eplerenone	80.4 ± 7.9	78.2 ± 9.3	76.3 ± 7.9	78.9 ± 9.9	0.11
Controls	79.2 ± 8.13	78.48 ± 8.39	76.44 ± 8.68	81.88 ± 9.75	0.019

**Table 6:** Serum potassium levels in patients treated with eplerenone and controls.

	Baseline Serum K^+^	Serum K^+^ 3 Months	Serum K^+^ 6 Months	Serum K^+^ 12 Months	*p*
Patients treated with eplerenone	4.45 ± 0.35	4.64 ± 0.36	4.51 ± 0.4	4.52 ± 0.37	0.12
Controls	4.47 ± 0.44	4.57 ± 0.53	4.52 ± 0.45	4.56 ± 0.51	0.49

## Data Availability

All anonymized data related to this work are available upon request to the corresponding author.

## Supplementary Materials

The following supporting information can be downloaded at https://www.mdpi.com/article/10.3390/biomedicines11123340/s1: Table S1: Differences of serum creatinine, eGFR, urine protein, SBP, DBP and K^+^ levels between groups at different time points.
