# Supplementary material for: A Prospective Study of Eplerenone in the Treatment of Patients with Glomerulonephritis
Source: Biomedicines. 2023 Dec 18;11(12):3340. doi: 10.3390/biomedicines11123340 (PMC10741749; doi:10.3390/biomedicines11123340)
Supplement: Supplementary file 1 [file biomedicines-11-03340-s001.zip › biomedicines-2729149-supplementary.pdf]

Supplemental Table S1. Differences of serum creatinine, eGFR, urine protein, SBP, DBP and K^+ levels between groups at different time points.

**Variable**	**Patients Treated with Eplerenone**	**Controls**	**p**
Serum creatinine (mg/dl ± SD)			
3 months	1.077 ± 0.29	1.296 ± 0.557	0.08
6 months	1.092 ± 0.28	1.197 ± 0.54	0.38
12 months	1.091 ± 0.3	1.292 ± 0.664	0.19
eGFR (ml/min/1.73m^2 ± SD)			
3 months	81.5 ± 29.5	65.4 ± 30.5	0.06
6 months	79 ± 27.2	69.5 ± 28.1	0.19
12 months	81 ± 32.1	66.6 ± 29.8	0.09
Urine protein (mg/24h ± SD)			
3 months	1555 ± 2083	1613 ± 2982	0.8
6 months	1237 ± 1491	1411 ± 1819	0.7
12 months	1152 ± 1363	1718 ± 2611	0.36
Systolic blood pressure (mmHg ± SD)			
3 months	126.5±14,5	129 ± 19	0.6
6 months	119.3±13	127.4 ± 22	0.12
12 months	125.2 ± 10.3	126 ± 16.1	0.87
Diastolic blood pressure (mmHg)			
3 months	78.2 ± 9.3	78.5 ± 8.4	0.91
6 months	76.3 ± 7.9	76.4 ± 8.7	0.94
12 months	78.9 ± 9.9	81.9 ± 9.75	0.3
Serum K^+ (meq/l)			
3 months	4.64 ± 0.36	4.57 ± 0.53	0.58
6 months	4.51 ± 0.4	4.52 ± 0.45	0.89
12 months	4.52 ± 0.37	4.56 ± 0.51	0.74
